# The Effect of Systemic Inflammation on Newborns: The Prognostic Value of the Aggregate Systemic Inflammation Index (AISI) and Systemic Inflammatory Response Index (SIRI)

**DOI:** 10.3390/diagnostics15121544

**Published:** 2025-06-17

**Authors:** Samet Kırat

**Affiliations:** Department of Gynecology and Obstetrics, Faculty of Medicine, Kafkas University, Kars 36000, Turkey; sametkirat1989@hotmail.com

**Keywords:** aggregate systemic inflammation index, inflammation, neonatal intensive care unit, preterm delivery, systemic inflammatory response index

## Abstract

**Objective:** This study aimed to investigate the prognostic value of two novel systemic inflammatory indices—the Aggregate Systemic Inflammation Index (AISI) and the Systemic Inflammatory Response Index (SIRI)—in predicting preterm delivery and associated neonatal outcomes. **Methods:** A retrospective, descriptive, cross-sectional study was conducted using the electronic health records of 1056 pregnant women admitted to a tertiary university hospital between 2020 and 2025. Pregnancies were classified into preterm (*n* = 528) and term (*n* = 528) groups. Demographic, obstetric, neonatal, and laboratory data were analyzed. **Results:** The AISI and SIRI values in the first trimester and at admission were significantly higher in the preterm delivery group than in the term delivery group (*p* < 0.001). Elevated AISI and SIRI levels correlated with lower 1st- and 5th-minute APGAR scores (*p* < 0.001) and higher neonatal intensive care unit (NICU) admission rates (35.8% vs. 4.5%; *p* < 0.001). The AISI cut-offs were 399.2 for preterm delivery (59.7% sensitivity, 59.8% specificity), 558.8 for NICU admission (79.3% sensitivity, 79.2% specificity), 694.0 for RDS (87.8% sensitivity, 87.8% specificity), 602.1 for sepsis (79.6% sensitivity, 79.2% specificity), and 753.8 for congenital pneumonia (81.6% sensitivity, 81.9% specificity). The SIRI cut-offs were 1.7 for preterm delivery (59.1% sensitivity, 58.9% specificity), 2.4 for NICU admission (81.7% sensitivity, 81.6% specificity), 3.1 for RDS (89.0% sensitivity, 89.5% specificity), 3.0 for sepsis (85.8% sensitivity, 85.7% specificity), and 3.4 for congenital pneumonia (85.7% sensitivity, 83.8% specificity). **Conclusions:** The AISI and SIRI showed significant predictive utility for neonatal morbidity in preterm delivery. The use of these markers in clinical practice may improve neonatal outcomes by enhancing the early diagnosis and management of high-risk pregnancies.

## 1. Introduction

Preterm delivery remains a leading cause of neonatal morbidity and mortality worldwide, contributing significantly to perinatal health burdens. The accurate prediction of preterm birth and related neonatal outcomes is essential for guiding timely clinical interventions and improving prognosis [1]. While various obstetric and fetal factors have been associated with preterm birth, increasing evidence suggests a prominent role of systemic inflammation in its pathophysiology.

Numerous inflammatory biomarkers, particularly C-reactive protein (CRP), interleukin-6 (IL-6), and tumor necrosis factor-alpha (TNF-α), have been investigated in relation to preterm delivery and neonatal complications. Systematic reviews have shown that elevated levels of these cytokines are associated with intrauterine infection, chorioamnionitis, and adverse neonatal outcomes such as sepsis and NICU admission [2,3,4]. However, their application in clinical settings is limited due to factors such as short half-lives, timing sensitivity, cost, and invasive sampling requirements. Additionally, simpler hematologic indices, such as the neutrophil-to-lymphocyte ratio (NLR) and platelet-to-lymphocyte ratio (PLR), have yielded inconsistent results and do not fully reflect the dynamic interactions among immune cell lines [5].

To overcome the various limitations of traditional inflammatory markers, which have been widely studied in relation to preterm delivery and neonatal complications, novel hematologic indices, such as the Aggregate Index of Systemic Inflammation (AISI) and the Systemic Inflammatory Response Index (SIRI), have been developed as non-invasive, easily accessible, and cost-effective alternatives that offer significant potential in clinical decision making [3,6]. These composite indices incorporate neutrophils, monocytes, lymphocytes, and platelets to reflect the severity of systemic inflammation from a broader immunological perspective [3,6,7]. Initially developed in oncology and cardiovascular research, the AISI and SIRI have shown predictive values in various inflammatory conditions [2,8,9,10]. Recent studies have begun to explore their potential in obstetric populations, particularly in the context of preterm birth and neonatal outcomes such as respiratory distress syndrome (RDS), neonatal sepsis, and NICU admission [3,11,12,13].

This study aimed to evaluate the performance of the AISI and SIRI in predicting preterm delivery, NICU admission, and major neonatal morbidities. We hypothesized that inflammatory index values would be significantly higher in women with preterm delivery and would serve as effective markers of neonatal prognosis. To the best of our knowledge, this is one of the first large-scale studies examining the role of the AISI and SIRI in the context of preterm delivery and associated neonatal outcomes. By analyzing these novel biomarkers in a large clinical cohort, we aim to support the development of practical, accessible, and non-invasive tools to enhance early risk stratification and improve perinatal care.

## 2. Materials and Methods

### 2.1. Patients and Data Collection

This retrospective, descriptive, and cross-sectional study was conducted on 1056 pregnant women admitted to the Obstetrics and Gynecology Clinic of a tertiary university hospital between 2020 and 2025. Women between the ages of 18 and 55 years with singleton pregnancies and complete blood count data for both the first trimester and the time of presentation were included in the study. Demographic and obstetric characteristics such as age, gravida, parity, abortion, smoking, employment status, educational level, socioeconomic status, and history of previous surgery were evaluated. Data such as gestational age, 1st- and 5th-minute APGAR scores of the newborn, history of NICU admission, the duration of NICU admission, and diagnosis at NICU admission (transient tachypnea of the newborn (TTN), respiratory distress syndrome (RDS), meconium aspiration syndrome (MAS), congenital pneumonia, and sepsis) were analyzed. The hemogram results of the pregnant women were evaluated at two time points: first, during routine antenatal screening in the first trimester (between 11 + 0 and 13 + 6 weeks of gestation), and second, at the time of hospital admission for delivery, regardless of gestational age. Neutrophil, lymphocyte, monocyte, and platelet values were recorded at both time points. All laboratory values were retrospectively retrieved from the hospital’s electronic health record (EHR) system.

### 2.2. Exclusion Criteria for the Study

Pregnant women under 18 years of age, women with chronic diseases, women with active urinary tract infections, women with active genital tract infections, women who conceived with assisted reproductive techniques, women with extrauterine pregnancies (e.g., ectopic), and women with multiple pregnancies were excluded from the study. These exclusion criteria were applied to obtain a more homogeneous study population and to minimize potential confounding factors that could affect inflammatory markers or pregnancy outcomes.

### 2.3. Group Selection

Pregnant women who had preterm deliveries (*n* = 528) constituted the study group, and those who had term deliveries in the same period (*n* = 528) constituted the control group. Firstly, within-group and control comparisons were made. Women who gave birth preterm were divided into three subgroups according to gestational age: early preterm (<32 + 0 weeks), middle preterm (32 + 0–33 + 6 weeks), and late preterm (34 + 0–36 + 6 weeks). The relationships between these groups were evaluated, and comparisons were made according to the parameters determined.

### 2.4. Systemic Inflammatory Response Index (SIRI)

A high SIRI is an indicator of severe inflammation and a weakened immune response in the body. The SIRI was first described to predict survival in pancreatic cancer patients [6] and has since been used as an important biomarker to assess the prognosis of many diseases in which inflammation plays a decisive role, such as cancer, cardiovascular diseases, and infections [8,9]. The SIRI is calculated as (neutrophils × monocytes)/lymphocytes and is considered a valuable clinical tool for predicting the course of diseases [6].

### 2.5. Aggregate Systemic Inflammation Index (AISI)

The AISI is a biomarker used to determine the level of systemic inflammation and is calculated using the formula (neutrophils × monocytes × platelets)/lymphocytes. High AISI values have been associated with clinical conditions that involve inflammatory processes, such as cancer, cardiovascular diseases, and autoimmune disorders. Unlike other inflammatory markers, this index integrates both myeloid and lymphoid cell populations, providing a more comprehensive and sensitive reflection of the systemic impact of inflammation [3].

### 2.6. Statistical Analysis

All statistical analyses were performed using IBM SPSS Statistics software (version 26.0; IBM Corp., Armonk, NY, USA). The chi-square (χ^2^) test was used to evaluate the relationships between categorical variables, and Fisher’s exact test was used for cells with few observations. In the comparison of continuous variables between the two groups, Student’s *t*-test and the Mann–Whitney U test were used for parametric and non-parametric data, respectively, depending on the normality of the distribution. In the comparison of three or more groups, appropriate analysis of variance was used for parametric data, while the Kruskal–Wallis H test was preferred for non-parametric data.

Stepwise linear regression analysis was applied to determine the factors associated with SIRI and AISI scores. In the first stage, univariate linear regression analyses were performed separately for each dependent variable, and statistically significant variables were determined. Then, only the significant variables were included in the multivariate linear regression model, and the final analysis was performed.

Logistic regression analysis was performed to evaluate the association of preterm delivery, NICU admission, TTN, RDS, RDS, MAS, sepsis, and congenital pneumonia with the AISI and SIRI. Based on the variables found to be significant in univariate analysis, multivariate logistic regression models were adjusted for potential confounders, such as maternal age, parity, smoking status, and socioeconomic level. Receiver operating characteristic (ROC) analysis curves were used to determine the diagnostic accuracy of the markers. Statistical significance was set at *p* < 0.05.

### 2.7. Ethics Committee Approval

This study was approved by the Non-Interventional Clinical Research Ethics Committee of the Kafkas University Faculty of Medicine (Approval Date: 10 February 2025, Decision No 80576354-050-99/615). This study was conducted in accordance with the ethical principles of the Declaration of Helsinki for Human Biomedical Research.

## 3. Results

### 3.1. Comparison of Preterm and Term Groups

Parity (*p* < 0.001) and the number of abortions (*p* < 0.001) were significantly higher in the term group. The smoking rate (36.9% vs. 14.8%; *p* < 0.001) and socioeconomic status (69.9% vs. 9.8%; *p* < 0.001) were significantly higher in the preterm group. The proportion of secondary school graduates was significantly higher in the term group (59.7% vs. 81.6%; *p* < 0.001). The preterm group had significantly lower 1st-minute APGAR (*p* < 0.001) and 5th-minute APGAR (*p* < 0.001) scores but significantly higher NICU admission rates (35.8% vs. 4.5%; *p* < 0.001) (Table 1).

In the preterm delivery group, SIRI levels at 1st trimester (1.4 (0–16) vs. 1.3 (0.10–26.5); *p* = 0.028), and time of admission (2.15 (0–16.4) vs. 1.56 (0.01–20.3); *p* < 0.001), and AISI levels were significantly higher in the 1st trimester (482.3 (0–3554.7) vs. 356.7 (3.1–4789.3); *p* < 0.001) (Table 1).

### 3.2. Comparison of Early Preterm, Middle Preterm, and Late Preterm Delivery Groups

The 1st-minute (*p* < 0.001) and 5th-minute APGAR scores (*p* < 0.001) were significantly lower in those who delivered in the early preterm period. The NICU admission rates (*p* < 0.001) and NICU admission durations (*p* < 0.001) were significantly higher in preterm delivery. The AISI and SIRI levels were significantly higher in the first trimester (both *p* < 0.001) and during admission (both *p* < 0.001) in preterm delivery (Table 2).

### 3.3. Receiver Operating Characteristic (ROC) Analysis Results

**Figure 1:**
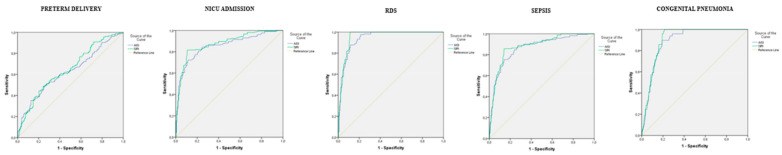
ROC analyses of AISI and SIRI indices for preterm delivery, NICU admission, RDS, sepsis, and congenital pneumonia.

The AISI had a moderate predictive power for preterm birth (AUC: 0.625) but showed strong discriminative performance for NICU admission (AUC: 0.855), RDS (AUC: 0.941), sepsis (AUC: 0.865), and congenital pneumonia (AUC: 0.892). It offered the best diagnostic accuracy with high sensitivity and specificity rates, especially for RDS (Table 3). The SIRI had higher predictive values for NICU admission (AUC: 0.878), RDS (AUC: 0.956), and congenital pneumonia (AUC: 0.905) compared to the AISI and showed the highest diagnostic accuracy for RDS (Table 3). These findings suggest that both indices have moderate predictive power for preterm delivery but may be important biomarkers for neonatal morbidities. The results of the ROC analysis of the AISI and SIRI for preterm delivery, NICU admission, RDS, sepsis, and congenital pneumonia are shown in Figure 1.

## 4. Discussion

In this study, we examined the prognostic value of the systemic inflammation indices AISI and SIRI in predicting preterm delivery and neonatal outcomes. According to our findings, both the AISI and SIRI levels were significantly higher in pregnant women with preterm delivery (*p* < 0.001). Furthermore, increased levels of these markers of inflammation were associated with a decrease in the 1st- and 5th-minute APGAR scores (*p* < 0.001) and a significant increase in NICU admission rates (*p* < 0.001). ROC analyses showed that the AISI and SIRI had strong discriminative value in predicting neonatal complications such as NICU admission, RDS, sepsis, and congenital pneumonia. These results suggest that indices of systemic inflammation may be important biomarkers for the early detection of preterm delivery and associated neonatal morbidities.

Studies have shown that the AISI has gained importance as a prognostic marker of inflammation and has been evaluated as a reliable biomarker in many clinical conditions, including perinatal morbidity. Elevated AISI levels have been associated with poor prognosis in acute myocardial infarction [2,8], severe disease and risk of death in COVID-19 [14,15,16], cardiovascular mortality in hypertension [10], high mortality risk in hemorrhagic stroke [17], disease progression in prostate and cervical cancers [9,18], and the highest mortality risk in patients receiving peritoneal dialysis [19]. It has been reported that AISI levels are higher in hypertensive pregnancies, and this may be associated with poor obstetric and neonatal outcomes [10]. In a study conducted in pregnant women with preterm premature rupture of membranes (PPROMs), the mean AISI was significantly higher (*p* < 0.05) in mothers of babies requiring NICU admission, in pregnant women with chorioamnionitis, and in those who delivered preterm (before 28 weeks: 945.6 vs. 604.9; before 34 weeks: 715.5 vs. 550.1) [3]. The cut-off values of the AISI were determined to be 626.1 for predicting NICU admission (74.1% sensitivity, 52.8% specificity), 506.0 for chorioamnionitis (68.9% sensitivity, 47.7% specificity), and 555.1 for delivery before 28 weeks (69.8% sensitivity, 48.1% specificity) [3].

In our study, increased AISI levels were linked to a higher preterm delivery rate (*p* < 0.001). In the preterm group, first-trimester AISI levels were significantly higher (482.3 [0–3554.7] vs. 356.7 [3.1–4789.3]; *p* < 0.001). Higher AISI levels correlated with lower 1st- and 5th-minute APGAR scores (*p* < 0.001), increased NICU admission rates (*p* < 0.001), longer admission durations (*p* < 0.001), and higher incidences of RDS (*p* < 0.001), sepsis (*p* < 0.001), and congenital pneumonia (*p* < 0.001). The AISI was associated with preterm delivery (OR: 1.001, 95% CI: 1.001–1.001, *p* < 0.001), NICU admission (OR: 1.003, 95% CI: 1.002–1.003, *p* < 0.001), RDS (OR: 1.003, 95% CI: 1.003–1.004, *p* < 0.001), sepsis (OR: 1.002, 95% CI: 1.002–1.003, *p* < 0.001), and congenital pneumonia (OR: 1.001, 95% CI: 1.001–1.002, *p* < 0.001). The AISI cut-offs were 399.2 for predicting preterm delivery (59.7% sensitivity, 59.8% specificity), 558.8 for NICU admission (79.3% sensitivity, 79.2% specificity), 694.0 for RDS (87.8% sensitivity, 87.8% specificity), 602.1 for sepsis (79.6% sensitivity, 79.2% specificity), and 753.8 for congenital pneumonia (81.6% sensitivity, 81.9% specificity). These results are consistent with prior studies reporting elevated AISI values in pregnancies with adverse neonatal outcomes [3]. Although the NICU-related cut-off reported in that study was somewhat higher than ours, the discrepancy may reflect variations in population characteristics, gestational ages, or sampling protocols. Taken together, these findings suggest that AISI may have potential as an accessible adjunctive marker in the early identification of high-risk pregnancies.

In general, the SIRI may play an important role in the prediction of obstetric and neonatal outcomes; however, it seems that the SIRI may provide more reliable results when combined with other biomarkers [20]. High SIRI levels may be significantly associated with preterm delivery, low birth weight, and NICU admission [12,20,21]. However, while some studies have suggested that high SIRI levels reduce the risk of RDS by promoting lung maturation, low SIRI levels have been reported to increase the risk of RDS, especially in infants born at <32 weeks [12,22]. It has also been shown to be a reliable marker for the diagnosis of neonatal early-onset sepsis [11]. Maternal inflammation has also been reported to increase the risk of neonatal systemic inflammatory response syndrome (SIRS) with high SIRI levels [23]. However, some studies suggest that the SIRI alone is not sufficient to predict preterm delivery and should be evaluated in conjunction with other inflammatory indices, such as the NLR and PLR [13].

In our study, increased SIRI levels significantly correlated with higher preterm delivery rates (*p* = 0.025). The SIRI was notably elevated in the preterm group during the first trimester (1.4 (0–16) vs. 1.3 (0.10–26.5); *p* = 0.028) and upon admission (2.1 (0–16.4) vs. 1.5 (0.01–20.3); *p* < 0.001). Higher SIRI levels corresponded with decreased 1st- and 5th-minute APGAR scores (*p* < 0.001), increased NICU admission rates (*p* < 0.001) and durations (*p* < 0.001), and higher incidences of RDS (*p* < 0.001), sepsis (*p* < 0.001), and congenital pneumonia (*p* < 0.001). The SIRI was linked to preterm delivery (OR: 1.063, 95% CI: 1.006–1.123, *p* = 0.029), NICU admission (OR: 2.096, 95% CI: 1.880–2.338, *p* < 0.001), RDS (OR: 1.809, 95% CI: 1.642–1.994, *p* < 0.001), sepsis (OR: 1.568, 95% CI: 1.441–1.707, *p* < 0.001), and congenital pneumonia (OR: 1.259, 95% CI: 1.157–1.371, *p* < 0.001). The SIRI cut-offs were 1.7 for predicting preterm delivery (59.1% sensitivity, 58.9% specificity), 2.4 for NICU admission (81.7% sensitivity, 81.6% specificity), 3.1 for RDS (89.0% sensitivity, 89.5% specificity), 3.0 for sepsis (85.8% sensitivity, 85.7% specificity), and 3.4 for congenital pneumonia (85.7% sensitivity, 83.8% specificity). These findings are broadly consistent with earlier reports linking elevated SIRI levels to adverse perinatal outcomes, including preterm birth, low birth weight, and NICU admission [12,20,21]. However, previous studies have also suggested that the predictive value of SIRI may improve when interpreted alongside other inflammatory indices such as NLR and PLR [13]. Therefore, while our results indicate a potential role for SIRI in early risk stratification, its integration into routine clinical practice requires further validation through prospective and multicenter studies.

Our study assesses the prognostic value of the systemic inflammation indices AISI and SIRI for preterm delivery and neonatal outcomes in a large sample. Measurements obtained in both the first trimester and at hospital admission strengthen the temporal association between systemic inflammation and adverse pregnancy outcomes. Subgroup analyses based on preterm birth severity, along with the exclusion of women with known chronic diseases and active infections, increase the internal validity of our findings. Nonetheless, the single-center and retrospective design limits its generalizability, and potential selection bias cannot be ruled out. Furthermore, although major confounders such as maternal age, parity, smoking, and socioeconomic status were adjusted for in multivariate models, the influence of undetected subclinical infections, nutritional status, psychological stress, or periodontal disease may have affected systemic inflammation and cannot be fully excluded. These factors may contribute to residual confounding and should be addressed in future prospective studies. Although the AISI and SIRI demonstrated promising predictive value, particularly for neonatal complications, they should still be regarded as investigational tools rather than established diagnostic markers. Further large-scale, multicenter, and prospective studies, including external validation, are required before these indices can be recommended for routine clinical use in obstetric care.

## 5. Conclusions

This study demonstrated the prognostic importance of systemic inflammation indices, particularly the AISI and SIRI, in predicting preterm delivery and neonatal outcomes. Our findings show that high AISI and SIRI levels are associated with increased preterm delivery risk, lower APGAR scores, higher NICU admission rates, and increased neonatal complications such as RDS, sepsis, and congenital pneumonia. While traditional inflammatory markers have been widely investigated, our study suggests that these novel hematologic indices can serve as accessible, cost-effective biomarkers for neonatal risk stratification. Despite the limitations of its single-center and retrospective design, this study provides important findings on the clinical value of the AISI and SIRI in obstetric and neonatal care. Future multicenter, prospective studies will help determine the value of these indices in clinical practice and their integration into perinatal risk assessment models.

## Figures and Tables

**Table 1:** Comparison of the preterm group and term group.

	Preterm Group (*n* = 528)	Term Group (*n* = 528)	*p*
**Age (years) (Median (Min–Max))**	33.5 (19–47)	34 (23–53)	0.534
**Gravida (Median (Min–Max))**	1 (1–6)	2 (1–6)	0.053
**Parity (Median (Min–Max))**	0 (0–4)	1 (0–4)	**<0.001**
**Abortus (Median (Min–Max))**	0 (0–3)	0 (0–3)	**<0.001**
**Smoking (*n*, %)**	195 (36.9%)	78 (14.8%)	**<0.001**
**Employment Status (*n*, %)**	195 (36.9%)	210 (39.8%)	0.342
**Education (*n*, %)**			
Primary school	144 (27.3%)	46 (8.7%)	**<0.001**
High school	315 (59.7%)	431 (81.6%)	**<0.001**
College/University	69 (13.1%)	51 (9.7%)	**<0.001**
**Yearly income level (*n*, %)**			
Low	27 (5.1%)	28 (5.3%)	**<0.001**
Medium	132 (25%)	448 (84.8%)	**<0.001**
High	369 (69.9%)	52 (9.8%)	**<0.001**
**Hospitalization Week (Median (Min–Max))**	34 (27–36)	39 (37–42)	**<0.001**
**1st minute APGAR score (Median (Min–Max))**	6 (2–7)	8 (7–9)	**<0.001**
**5th minute APGAR score (Median (Min–Max))**	8 (5–9)	9 (8–10)	**<0.001**
**NICU (*n*, %)**	189 (35.8%)	24 (4.5%)	**<0.001**
RDS	164 (31.1%)	0 (0%)	**<0.001**
Bacterial sepsis	146 (27.7%)	16 (3%)	**<0.001**
TTN	21 (4%)	13 (2.5%)	0.222
MAS	8 (1.5%)	6 (1.1%)	0.788
Congenital Pneumonia	49 (9.3%)	0 (0%)	**<0.001**
**Duration of NICU Hospitalization** **(Median (Min–Max))**	0 (0–28)	0 (0–10)	**<0.001**
**SIRI (Median (Min–Max))**			
1st trimester	1.4 (0–16)	1.3 (0.1–26.5)	**0.028**
Hospitalization Time	2.1 (0–16.4)	1.5 (0.01–20.3)	**<0.001**
**AISI (Median (Min–Max))**			
1st trimester	482.3 (0–3554.7)	356.7 (3.1–4789.3)	**<0.001**
Hospitalization Time	310.8 (0–3136)	306.8 (5.2–5981.8)	0.545

**Table 2:** Comparison of early preterm, middle preterm, and late preterm delivery groups.

	Early Preterm (*n* = 78)	Middle Preterm (*n* = 111)	Late Preterm (*n* = 339)	*p*
**Age (years) (Median (Min–Max))**	34 (24–44)	34 (23–46)	33 (19–47)	0.667
**Gravida (Median (Min–Max))**	1 (1–4)	1 (1–5)	1 (1–6)	0.597
**Parity (Median (Min–Max))**	0 (0–3)	0 (0–3)	0 (0–4)	0.935
**Abortus (Median (Min–Max))**	0 (0–1)	0 (0–3)	0 (0–2)	0.655
**Smoking (*n*, %)**	21 (26.9%)	36 (32.4%)	138 (40.7%)	**0.041**
**Employment Status (*n*, %)**	24 (30.8%)	27 (24.3%)	144 (42.5%)	**0.001**
**Education (*n*, %)**				
Primary school	18 (23.1%)	12 (10.8%)	114 (33.6%)	**<0.001**
High school	51 (65.4%)	75 (67.6%)	189 (55.8%)	**<0.001**
College/University	9 (11.5%)	24 (21.6%)	36 (10.6%)	**<0.001**
**Yearly income level (*n*, %)**				
Low	0 (0%)	12 (10.8%)	15 (4.4%)	**<0.001**
Medium	15 (19.2%)	9 (8.1%)	108 (31.9%)	**<0.001**
High	63 (80.8%)	90 (81.1%)	216 (63.7%)	**<0.001**
**Hospitalization Week (Median (Min–Max))**	30 (27–31)	32 (32–33)	35 (34–36)	**<0.001**
**1st minute APGAR score (Median (Min–Max))**	2 (2–3)	5 (4–6)	6 (6–7)	**<0.001**
**5th minute APGAR score (Median (Min–Max))**	6 (5–7)	7 (6–8)	8 (8–9)	**<0.001**
**NICU (*n*, %)**	78 (100%)	90 (81.1%)	21 (6.2%)	**<0.001**
RDS	78 (100%)	86 (77.5%)	0 (0%)	**<0.001**
Bacterial sepsis	63 (80.8%)	72 (64.9%)	11 (3.2%)	**<0.001**
TTN	0 (0%)	0 (0%)	21 (6.2%)	**0.002**
MAS	0 (0%)	0 (0%)	8 (2.4%)	0.104
Congenital Pneumonia	23 (29.5%)	26 (23.4%)	0 (0%)	**<0.001**
**Duration of NICU Hospitalization** **(Median (Min–Max))**	15 (7–28)	7 (0–7)	0 (0–10)	**<0.001**
**SIRI (Median (Min–Max))**				
1st trimester	5.7 (4.0–16)	2.7 (1.8–4.3)	0.9 (0–2.3)	**<0.001**
Hospitalization Time	6.8 (5.2–16.4)	3.5 (3.0–4.9)	1.4 (0–2.9)	**<0.001**
**AISI (Median (Min–Max))**				
1st trimester	1571 (576–3554)	857 (460–1680)	302 (0–952)	**<0.001**
Hospitalization Time	1219 (354–3136)	612 (236–1371)	165 (0–674)	**<0.001**

**Table 3:** Receiver operating characteristic (ROC) analysis for preterm delivery, NICU hospitalization, RDS, sepsis, and congenital pneumonia.

Variables	AUC	S.E.	*p*	OR (95% CI)	Cutt-Off	Sensitivity	Specificity
**Preterm Delivery**							
AISI	0.625	0.017	**<0.001**	0.592–0.659	399.2	59.7%	59.8%
SIRI	0.648	0.017	**<0.001**	0.615–0.680	1.7	59.1%	58.9%
**NICU**							
AISI	0.855	0.016	**<0.001**	0.823–0.886	558.8	79.3%	79.2%
SIRI	0.878	0.014	**<0.001**	0.851–0.906	2.4	81.7%	81.6%
**RDS**							
AISI	0.941	0.007	**<0.001**	0.927–0.955	694	87.8%	87.8%
SIRI	0.956	0.006	**<0.001**	0.944–0.967	3.1	89%	59.5%
**Sepsis**							
AISI	0.865	0.016	**<0.001**	0.833–0.896	602.1	79.6%	79.2%
SIRI	0.822	0.014	**<0.001**	0.854–0.910	3	85.8%	85.7%
**Congenital Pneumonia**							
AISI	0.892	0.015	**<0.001**	0.863–0.920	753.8	81.6%	81.9%
SIRI	0.905	0.011	**<0.001**	0.883–0.927	3.4	85.7%	83.8%

**AUC:** Area Under the Curve, **AISI:** Aggregate Index of Systemic Inflammation, **NICU:** Neonatal Intensive Care Unit, **OR:** Odds Ratio, ***p*:** *p*-value, **RDS:** Respiratory Distress Syndrome, **S.E.:** Standard Error, **SIRI:** Systemic Inflammatory Response Index.

## Data Availability

All data related to the study have been included in the article.

## Abbreviations

AISI	Aggregate Index of Systemic Inflammation
MAS	Meconium Aspiration Syndrome
NICU	Neonatal Intensive Care Unit
NLR	Neutrophil-to-Lymphocyte Ratio
PLR	Platelet-to-Lymphocyte Ratio
PPROM	Preterm Premature Rupture of Membranes
RDS	Respiratory Distress Syndrome
ROC	Receiver Operating Characteristic
SIRI	Systemic Inflammatory Response Index
SIRS	Systemic Inflammatory Response Syndrome
TTN	Transient Tachypnea of the Newborn
